# Supplementary material for: Integrated environmental and clinical surveillance for the prevention of acute respiratory infections (ARIs) in indoor environments and vulnerable communities (Stell-ARI): Protocol
Source: PLoS One. 2024 Sep 30;19(9):e0309111. doi: 10.1371/journal.pone.0309111 (PMC11441648; doi:10.1371/journal.pone.0309111)
Supplement: Attachment — Submitted filename: Study Protocol - PNRR-UNIPI Response to Reviewers .docx [file pone.0309111.s001.docx]

**Integrated environmental and clinical surveillance for the prevention of acute respiratory infections (ARIs) in indoor environments and vulnerable communities (Stell-ARI): Protocol**

**Rebuttal Letter**

Dear Sara Hemati,

We appreciate the opportunity to revise our manuscript titled “Integrated Environmental and Clinical Surveillance for the Prevention of Acute Respiratory Infections (ARIs) in Indoor Environments and Vulnerable Communities (Stell-ARI): Protocol.” In this revision, we have thoughtfully considered the suggestions provided by both the editor and the reviewers, making adjustments and additions accordingly. We addressed each point raised, starting with the reviewers’ comments followed by those from the editor. We are grateful for the constructive feedback on our original submission and believe that the changes have significantly enhanced the quality of the paper.

Best regards,

Atomsa Nebiyu Tariku

**Responses to reviewer #1:**

‎

1. “Discussion” section should be revised basically. It is not suitable for a manuscript. ‎The ‎manuscript needs more adequate discussion with supporting latest references.

We have carefully reviewed your comments and have substantially revised this section to enhance its suitability for the manuscript. We have expanded our discussion to provide a more comprehensive analysis and have included additional references from the latest literature to support our points. We believe these revisions address the concerns raised and improve the overall quality and depth of the discussion. The changes made were the following:

The following section was added to the manuscript:

To face the risk of ARIs outbreak onset and spreading in closed communities, different interventions can be adopted according to the natural history of the disease:

- Before a pathogen enters the community, assessing environmental conditions (using environmental questionnaires) and contamination (through surface and air monitoring), along with understanding people’s behaviours and health status (via basal questionnaires), can facilitate a preliminary risk assessment. Quantitative Microbial Risk Assessment (QMRA) models may be employed for this purpose. The results of this assessment can guide the adoption of preventive measures, even in the absence of the specific pathogen. These measures may include cleaning, ventilation, masks usage, and vaccination, The outcomes of the STELL-ARI project can identify proxies, indicators and index pathogens for an efficient, reliable, and sustainable continuous risk assessment, allowing the establishment of thresholds to recommend appropriate interventions.
- When a pathogen is first introduced into a community, efficient outbreak control relies on timely detection, ideally before symptomatic cases emerge. An effective early warning system can be established using wastewater-based surveillance (WBS), Which has already been extensively used for SARS-CoV-2 in communities [44]. The STELL-ARI project can demonstrate the potential application of this surveillance method for other respiratory viruses as well. Once an early warning signal is detected, reinforcing preventive measures becomes crucial. After the outbreak, the WBS can indicate the cessation of viral circulation.
- Finally, clinical and virological surveillance within communities enables the monitoring of symptoms and clinical outcomes, facilitating an evaluation of disease epidemiology and the effectiveness of preventive measures.
- Overall, the project aims to establish a feasible and cost-effective approach to addressing uncertainties surrounding future viral outbreaks. By enabling rapid response and preparedness [45, it might provide, even with limited resources and time [46], an effective methodology for evaluating an acute viral spread and a long-term surveillance [47]. Numerous studies demonstrate the efficacy of predictive modelling that integrates clinical data and wastewater monitoring for tracking respiratory viruses’ spread within communities and anticipating outbreaks [48, 49]. However, there is limited data on the potential combination of these predictive models with quantitative microbial risk assessment (QMRA), particularly in closed and vulnerable communities. QMRA offers a structured approach to evaluate the risk posed by infectious agents in various settings [50], but its integration with near-real-time surveillance systems remains underexplored. By combining predictive modelling with QMRA, it may be possible to identify and mitigate risks in these environments, ultimately leading to more effective prevention and control measures. (Pag 9, line 487-519 of the revised “Manuscript”)

The following sections in the discussion section of the manuscript were modified:

| **Previous version** | **Revised version** (Pag. 9, line 484-486) |
| --- | --- |
| “By integrating clinical, behavioural and environmental data, it may be possible to provide the authorities with specific information for early detection and mitigation of ARIs.” | “By integrating environmental, behavioural and clinical data, it may be possible to provide the authorities with specific information for early detection and mitigation of ARIs.” |
| **Previous version** | **Revised version** (Pag. 10, line 519-525) |
| “The study's emphasis on predictive modelling and risk assessment may allow to detect and respond to outbreaks before they reach critical levels, thus minimising their impact. Since this study involves different settings such as LTCFs, prisons and primary schools, it enhances the study's relevance and transferability across a spectrum of closed settings characterized by very different living conditions and population socio-demographic composition.” | “The study's emphasis on combining predictive modelling with risk assessment could enhance our ability to detect and respond to outbreaks in these environments before they reach critical levels, thus minimising their impact. Since this study involves different settings such as LTCFs, prisons and primary schools, it enhances the study's relevance and transferability across a spectrum of closed settings characterized by very different living conditions and population socio-demographic composition.” |

The following references has been added to this section to support our argument:

Zhao L, Zou Y, David RE, Withington S, McFarlane S, Faust RA, et al. Simple methods for early warnings of COVID-19 surges: Lessons learned from 21 months of wastewater and clinical data collection in Detroit, Michigan, United States. Sci Total Environ. 2023;864(161152):161152.

Kamalrathne T, Amaratunga D, Haigh R, Kodituwakku L. Need for effective detection and early warnings for epidemic and pandemic preparedness planning in the context of multi-hazards: Lessons from the COVID-19 pandemic. Int J Disaster Risk Reduct. 2023;92(103724):103724.

Maryam S, UI Haq I, Yahya G, UI Haq M, Algammal AM, Saber S, et al. COVID-19 surveillance in wastewater: An epidemiological tool for the monitoring of SARS-CoV-2. Front Cell Infect Microbiol. 2022;12:978643.

Hewitt J, Trowsdale S, Armstrong BA, Chapman JR, Carter KM, Croucher DM, et al. Sensitivity of wastewater-based epidemiology for detection of SARS-CoV-2 RNA in a low prevalence setting. Water Res. 2022;211(118032):118032.

Shah S, Gwee SXW, Ng JQX, Lau N, Koh J, Pang J. Wastewater surveillance to infer COVID-19 transmission: A systematic review. Sci Total Environ. 2022;804(150060):150060.

Torabi F, Li G, Mole C, Nicholson G, Rowlingson B, Smith CR, et al. Wastewater-based surveillance models for COVID-19: A focused review on spatio-temporal models. Heliyon. 2023;9(11):e21734.

Carducci A, Donzelli G, Cioni L, Federigi I, Lombardi R, Verani M. Quantitative Microbial Risk Assessment for Workers Exposed to Bioaerosol in Wastewater Treatment Plants Aimed at the Choice and Setup of Safety Measures. Int J Environ Res Public Health. 2018;15(7):1490.

2. In the discussion section, the potential limitation of the study should be highlighted and ‎in ‎the conclusion, novel insight should be clearly highlighted.‎ ‎

Thank you for your constructive feedback. In response to your comments, we have expanded the "Discussion" section to include a detailed analysis of the potential limitations of our study. Additionally, we have clearly highlighted the novel insight of our research, specifically the integration of clinical, behavioral and environmental data and the combination of predictive modeling with quantitative microbial risk assessment (QMRA) to limit and prevent the spread of ARI’s. We believe these enhancements provide a more comprehensive overview of our study's implications and innovative contributions. Specifically we have made the following changes:

- The description of the vulnerability of the community has been modified as in the table below:

| **Previous version** | **Revised version** (Pag. 10, line 527-532) |
| --- | --- |
| “A complete correlation between clinical and environmental data could not be representative in case of limited data collection for insufficient number of study subjects providing the informed consent (e.g. refusal to undergo to nasopharyngeal swabs) or higher than expected loss to follow-up. This study potentially demands sustained participant engagement and ongoing data collection, which may discourage prospective participants, especially if in situation of vulnerability, as well as parents of school-children.” | “A complete correlation between clinical and environmental data could not be representative in case of limited data collection due to insufficient number of study subjects providing informed consent (e.g. refusal to undergo to nasopharyngeal swabs) or higher than expected loss to follow-up. This study potentially demands sustained participant engagement and ongoing data collection, which may discourage prospective participants, especially those in vulnerable situation, as well as parents of school children.” |

- The following paragraph has been added to the limitation section of the discussion:

“Additionally, privacy concerns related to individual data collection and management, especially in a school environment, may reduce participants’ confidentiality and trust. Another limitation lies in the large variability of environmental conditions and individual behaviours across the different settings analysed, potentially affecting the generalizability of the findings to other context. Finally, while innovative, reliance on advanced analysis techniques and biomolecular PCR tests could pose challenges in terms of expertise required for implementing this approach across various setups.

” (Pag. 11, line 532-537 of the revised “Manuscript”)

- The following paragraph was modified and an additional reference was inserted, namely the added reference was:

Maida CM, Amodio E, Mazzucco W, La Rosa G, Lucentini L, Suffredini E, et al. Wastewater-based epidemiology for early warning of SARS-COV-2 circulation: A pilot study conducted in Sicily, Italy. Int J Hyg Environ Health. 2022;242(113948):113948

| **Previous version** | **Revised version** (Pag. 11, line 538-587) |
| --- | --- |
| “However, there are also several strengths. Since Stell-ARI’s approach integrates clinical, behavioural, and environmental data, it enhances the accuracy and effectiveness of ARIs surveillance, providing a more complete understanding of transmission dynamics. The combination of predictive models and risk assessment tools, facilitates early warning systems and minimises disease spread.” | However, there are also several strengths. Since Stell-ARI’s approach integrates environmental, behavioural, and clinical data, it enhances the accuracy and effectiveness of ARIs surveillance by capturing a multifaceted view of the factors influencing viral spread and providing a more complete understanding of transmission dynamics. This approach is valuable for identifying high-risk areas and populations, enabling continuous observation and rapid responses to emerging threats. It also facilitates early warning systems, proper resource allocation, and minimization of disease spread [51]. |

- The following statement was added to provide more substance to the strength of our study:

“Tailoring interventions based on specific risk factors identified through comprehensive data analysis may lead to more effective prevention and control measures. Additionally, including behaviouralnto the risk assessment tool provides insights into the impact of human actions and social behaviours on ARI transmission, aiding in the design of effective public health measures to reduce disease spread. The implementation of this comprehensive approach in challenging settings, such as prisons, schools, and long-term care facilities - where data collection is particularly difficult and scarce literature exists - adds value to the Stell-ARI project. Furthermore, the reproducibility of this integrated system into other settings is also a significant strength, since it can be adapted to various environments, including long-term care facilities, dormitories, schools, and prisons.” (Pag. 11, Line 543-552 of the revised “Manuscript”)

- The aspect of the limitation section has been improved as reported below:

| **Previous version** | **Modified version** (Pag. 12, line 552-554) |
| --- | --- |
| “In addition, Stell-ARI ensures participants privacy, respecting informed consent and pseudonymized data collection. This commitment will attest the study's credibility and minimise ethical concerns.” | “In addition, Stell-ARI ensures participants privacy, respecting informed consent and pseudonymized data collection. This commitment will attest the study's credibility and minimise ethical concerns, encouraging participants in continued engagement in surveillance efforts.” |

3. It is also recommended to discuss and explain what should be the appropriate policies based ‎on the findings of this literature.‎

Thank you for your valuable feedback. In response to your suggestion, we have expanded the discussion section to include a detailed explanation of appropriate policies based on our findings. Specifically, we recommend enhancing public health strategies by integrating data collection from various sources, implementing early warning systems in closed and vulnerable communities, and focusing on environmental and behavioral factors to improve indoor air quality and reduce crowding.

These policy recommendations are outlined in the newly added section to ensure clarity and comprehensiveness as follows:

“The approach of the Stell-ARI project may offer several policy recommendations to enhance public health strategies and reduce ARIs. First, integrating data from various sources, such as clinical, behavioral, and environmental data, can facilitate the timely provision of real-time information to decision-makers. Second, implementing early warning systems in closed and vulnerable communities can help limit potential outbreaks by enabling rapid response and containment measures. Third, considering environmental and behavioural factors in the transmission and prevention of ARIs can encourage policymakers to prioritize public health strategies that improve indoor air quality. This might be through better ventilation, regular monitoring of enclosed spaces, and measures to reduce crowding in confined spaces.” (Pag. 12, Line 555-562 of the revised “Manuscript”)

4. Please make sure your conclusions and future perspectives section underscores the ‎scientific value-added of your paper and/or the applicability of your results. ‎

Thank you for your feedback. We have updated the final section highlighting how our holistic approach addresses critical gaps in current public health strategies and provides actionable policy recommendations to enhance surveillance, early warning systems, and indoor air quality, ultimately improving public health outcomes. We have added in particular the following statement:

“This comprehensive approach, not only adds scientific value and practical applicability in the field of ARI prevention by addressing critical gaps and deficiencies, but also serves as a potential model for public health policy interventions.” (Pag. 12, Line 564-567 of the revised “Manuscript”)

**Responses to reviewer #2:**

1. I have thoroughly enjoyed reading your manuscript and believe it holds significant potential for publication. Your detailed methodology, clear objectives, and comprehensive approach to integrating various data streams are truly commendable. This study has the potential to greatly enhance our understanding and management of ARIs in enclosed environments, making a valuable contribution to public health.

Congratulations on your excellent work, and thank you for your valuable contribution to the field. I have no additional comments or suggestions for improvement. I highly recommend this manuscript for publication.

Thank you very much for your thoughtful and encouraging comments on our manuscript. Your feedback is greatly appreciated, and we are thrilled to hear that you found our study promising for publication. Your recognition of our methodology, objectives, and approach means a lot to us. We sincerely appreciate the time you dedicated to reading and evaluating our manuscript. Your thorough review and positive assessment are truly motivating. We are grateful for your confidence in our work and your recommendation for publication.

**Responses to Editor’s comments:**

1. Please ensure that your manuscript meets PLOS ONE's style requirements, including those for file naming. The PLOS ONE style templates can be found at: http://www.journals.plos.org/plosone/s/file?id=wjVg/PLOSOne_formatting_sam ple_main_body.pdf http://www.journals.plos.org/plosone/s/file?id=ba62/PLOSOne_formatting_sam ple_title_authors_affiliations.

Thank you for your feedback. We have reviewed and made the necessary modifications to the figure and table citations as indicated in the guidelines. As recommended, the figures are saved in the accepted formats and uploaded separately. Additionally, we have corrected the in-text citations by using the Vancouver format, placing the numbers into brackets.

2. Thank you for stating the following in the Acknowledgments Section of your manuscript: "The authors acknowledge support from the Lagrange Project of the Institute for Scientific Interchange Foundation (ISI Foundation) funded by Fondazione Cassa di Risparmio di Torino (Fondazione CRT). DP was supported by the VERDI project (101045989), funded by the Horizon Europe programme of the European Union. Views and opinions expressed are however those of the author(s) only and do not necessarily reflect those of the European Union or the Health and Digital Executive Agency. Neither the European Union nor the granting authority can be held responsible for them. LD and CC were supported by Fondation Botnar." We note that you have provided funding information that is not currently declared in your Funding Statement. However, funding information should not appear in the Acknowledgments section or other areas of your manuscript. We will only publish funding information present in the Funding Statement section of the online submission form. Please remove any funding-related text from the manuscript and let us know how you would like to update your Funding Statement. Currently, your Funding Statement reads as follows: "This project is funded by the National Government of Italy through the National Recovery and Resilience Plan (NRRP) 'Italy Tomorrow', which is part of the Next Generation EU (NGEU) programme. The NRRP aims to address the economic and social impacts of the SARS-CoV-2 pandemic crisis while addressing structural weaknesses in the Italian economy and promoting ecological and environmental transition. The Tuscany Health Ecosystem (THE), coordinated by the University of Florence, aligns with the NRRP's focus on 'Life Sciences', fostering growth in Tuscany's life sciences sector through collaboration between public and private entities to address innovation needs. THE comprises 10 Spokes, with our study falling under Spoke 2 (Preventive and Predictive Medicine), led by the University of Pisa's Department of Biology, in collaboration with other departments. In accordance with the NRRP, the Research and Innovation Programme "THE - Tuscany Health Ecosystem" has a 36-month eligibility period, commencing on 01/12/2022 and extending no later than 28/02/2026. Investment Line 1.5 allocates funding for the creation and/or strengthening of 12 Innovation Ecosystems (THE) across the national territory. This specific project falls under Spoke 2 (Preventive and Predictive Medicine), with project code I53C22000780001 with a budget of 1,308,657.86 €. The funders (The Italian Government) had no involvement in the study design, data collection and analysis, decision to publish, or manuscript preparation." Please include your amended statements within your cover letter; we will change the online submission form on your behalf.

Thank you for your comment. The section in question pertains to a collaboration with the ISI Foundation, focusing on the contact tracing aspect of the study within the school community and the handling of the online registration Influweb platform. This collaboration is not a direct funding source for the project. However, we understand the importance of transparency and have therefore added this section to the funding section to avoid any potential confusion. Additionally, we have removed any funding related statement from the manuscript and revised the acknowledgment section as follows:

| **Previous version** | **Revised version** (Pag. 14, Line 664-669) |
| --- | --- |
| “We thank all the staff of the enrolled settings for their cooperation in the implementation of the project: LTCFs (Alberto Meneghini, Maria Teresa Carracino, Giulia Del Punta, Elena Brugiati), prisons (Anna Santinami, Lisa Perugino, Santina Savoca) and primary schools (Rossana Condello, Germana Delle Canne, Claudia Bertani, Valeria Di Natale). We thank ISI foundation for their support providing Influweb platform and developing a dedicated part of the platform for our study: Giovanni Frigione and Matteo Delfino. The authors acknowledge support from the Lagrange Project of the Institute for Scientific Interchange Foundation (ISI Foundation) funded by Fondazione Cassa di Risparmio di Torino (Fondazione CRT). DP was supported by the VERDI project (101045989), funded by the Horizon Europe programme of the European Union. Views and opinions expressed are however those of the author(s) only and do not necessarily reflect those of the European Union or the Health and Digital Executive Agency. Neither the European Union nor the granting authority can be held responsible for them. LD and CC were supported by Fondation Botnar.” | "We thank all the staff of the enrolled settings for their cooperation in the implementation of the project: LTCFs (Alberto Meneghini, Maria Teresa Carracino, Giulia Del Punta, Elena Brugiati), prisons (Enrico Della Porta, Anna Santinami, Lisa Perugino, Santina Savoca), and primary schools (Rossana Condello, Germana Delle Canne, Claudia Bertani, Valeria Di Natale). We thank the ISI Foundation for their support in providing the Influweb platform and developing a dedicated part of the platform for our study: Giovanni Frigione and Matteo Delfino." |

3. Please review your reference list to ensure that it is complete and correct. If you have cited papers that have been retracted, please include the rationale for doing so in the manuscript text, or remove these references and replace them with relevant current references. Any changes to the reference list should be mentioned in the rebuttal letter that accompanies your revised manuscript. If you need to cite a retracted article, indicate the article’s retracted status in the References list and also include a citation and full reference for the retraction notice.

Thank you for your comment. We have diligently reviewed and updated the reference list to ensure its completeness and accuracy. We have checked each reference for correctness and relevance. We confirm that no retracted papers were present in our reference list. These updates have been reflected in the revised manuscript. We’ve added the following 7 additional references in the manuscript and formatted them in Vancouver style:

Zhao L, Zou Y, David RE, Withington S, McFarlane S, Faust RA, et al. Simple methods for early warnings of COVID-19 surges: Lessons learned from 21 months of wastewater and clinical data collection in Detroit, Michigan, United States. Sci Total Environ. 2023;864(161152):161152.

Kamalrathne T, Amaratunga D, Haigh R, Kodituwakku L. Need for effective detection and early warnings for epidemic and pandemic preparedness planning in the context of multi-hazards: Lessons from the COVID-19 pandemic. Int J Disaster Risk Reduct. 2023;92(103724):103724.

Maryam S, UI Haq I, Yahya G, UI Haq M, Algammal AM, Saber S, et al. COVID-19 surveillance in wastewater: An epidemiological tool for the monitoring of SARS-CoV-2. Front Cell Infect Microbiol. 2022;12:978643.

Hewitt J, Trowsdale S, Armstrong BA, Chapman JR, Carter KM, Croucher DM, et al. Sensitivity of wastewater-based epidemiology for detection of SARS-CoV-2 RNA in a low prevalence setting. Water Res. 2022;211(118032):118032.

Shah S, Gwee SXW, Ng JQX, Lau N, Koh J, Pang J. Wastewater surveillance to infer COVID-19 transmission: A systematic review. Sci Total Environ. 2022;804(150060):150060.

Torabi F, Li G, Mole C, Nicholson G, Rowlingson B, Smith CR, et al. Wastewater-based surveillance models for COVID-19: A focused review on spatio-temporal models. Heliyon. 2023;9(11):e21734.

Carducci A, Donzelli G, Cioni L, Federigi I, Lombardi R, Verani M. Quantitative Microbial Risk Assessment for Workers Exposed to Bioaerosol in Wastewater Treatment Plants Aimed at the Choice and Setup of Safety Measures. Int J Environ Res Public Health. 2018;15(7):1490.

Maida CM, Amodio E, Mazzucco W, La Rosa G, Lucentini L, Suffredini E, et al. Wastewater-based epidemiology for early warning of SARS-COV-2 circulation: A pilot study conducted in Sicily, Italy. Int J Hyg Environ Health. 2022;242(113948):113948.

Thank you again for your time and consideration,

Best regards

Atomsa Nebiyu Tariku, on behalf of STELL-ARI project
